# Recurrent back pain during working life and exit from paid employment: a 28-year follow-up of the Whitehall II Study

**DOI:** 10.1136/oemed-2018-105202

**Published:** 2018-10-04

**Authors:** Tea Lallukka, Minna Mänty, Cyrus Cooper, Maria Fleischmann, Anne Kouvonen, Karen E Walker-Bone, Jenny A Head, Jaana I Halonen

**Affiliations:** 1 Population and Work Ability Program, Finnish Institute of Occupational Health, Helsinki, Finland; 2 Department of Public Health, University of Helsinki, Helsinki, Finland; 3 Department of Research, Development and Innovation (RDI), Laurea University of Applied Sciences, Vantaa, Finland; 4 MRC Lifecourse Epidemiology Unit, University of Southampton, Southampton, UK; 5 NIHR Musculoskeletal Biomedical Research Unit, University of Oxford, Oxford, UK; 6 Department of Epidemiology and Public Health, University College London, London, UK; 7 Faculty of Social Sciences, University of Helsinki, Helsinki, Finland; 8 Division of Health Psychology, SWPS University of Social Sciences and Humanities in Wroclaw, Wroclaw, Poland; 9 UKCRC Centre of Excellence for Public Health, Queen’s University Belfast, Belfast, UK; 10 Arthritis Research UK/MRC Centre for Musculoskeletal Health and Work, University of Southampton, Southampton, UK

**Keywords:** back disorders, epidemiology, pain, employment transitions, occupational cohort

## Abstract

**Objectives:**

To examine the impact of recurrent, as compared with single, reports of back pain on exit from paid employment over decades of follow-up.

**Methods:**

The study sample was from the British Whitehall II Study cohort (n=8665, 69% men, aged 35–55 at baseline), who had provided information about their reports of back pain between 1985 and 1994. Data about exit from paid employment (health-related and non-health related exit, unemployment and other exit) were collected between 1995 and 2013. Repeated measures logistic regression models were fitted to examine the associations, and adjust for covariates.

**Results:**

Recurrent pain was reported by 18% of participants, while 26% reported pain on an occasion and 56% did not report pain. Report of back pain on an occasion was not associated with health-related job exit, whereas recurrent pain was associated with such an exit (OR 1.51; 95% CI 1.15 to 1.99), when compared with those who did not report pain. These associations were somewhat stronger among middle-grade and lower-grade employees, while these associations were not seen among higher-grade employees. Differences in associations by age and psychosocial working conditions were small.

**Conclusions:**

These results highlight the need for early detection of recurrent back pain to prevent exit out of paid employment for health reasons. As the risk varies by occupational grade, this emphasises the importance of identification of high-risk groups and finding ways to address their modifiable risk factors.

**Key messages**

**What is already known about this subject?**

- Low back pain is a recognised risk factor for work disability but studies have typically only explored associations between a baseline pain assessment and a follow-up event.
- Few studies have enabled the assessment of the impact of recurrent pain, as compared with single reports, especially collected prospectively over decades of follow-up.

**What are the new findings?**

- We have shown that recurrent, but not single reports of low back pain, predict health-related work exit, over decades of follow-up with trends that are more important in low-grade and middle-grade workers.
- We found that psychosocial working conditions were not effect modifiers in these associations.
- This study covered all major routes of exit and our findings strengthen the evidence about the public health and societal consequences of recurrent low back pain among employees during a working life span.

**How might this impact on policy or clinical practice in the foreseeable future?**

- Strategies to identify and prevent recurrent back pain could reduce the burden of musculoskeletal disorders on work disability.

## Introduction

Low back pain and other musculoskeletal disorders are among the leading causes of work disability, sickness absence and early exit from paid employment. Societal burden and cost of pain are notable.^1^ Acute, chronic and multisite pain have been associated with a risk of work disability.^2 3^ However, pain may contribute to exit out of paid employment in a number of ways, perhaps as a cause of health-related exit and as a contributing factor in exit due to unemployment.^4^ Thus, to get a full picture, different routes of exit from paid employment should be distinguished.

Although pain is linked to the risk of exit from work, it is unknown if the association is attributable to social factors predicting both pain and the exit. Pain is more prevalent among people with adverse socioeconomic circumstances,^5 6^ and it has been previously shown that parental socioeconomic position is associated with low back disorders in mid-life.^7^ The latter highlights the importance of the life course epidemiology when investigating the determinants and consequences of pain^8–10^ particularly as working conditions^11–13^ and behaviour-related risk factors, such as obesity,^14–16^ are associated with pain.

A notable limitation of the available research is that back pain, even chronic pain, has been assessed at a time point only. Based on a single measurement, it cannot be concluded whether it is the single report of pain that is the significant risk factor for exit out of paid employment, or whether such associations are in fact reflecting recurrent reports of pain. It is well known that low back pain can be recurrent and that future low back pain is associated with past low back pain but it is still important to understand the impact of both patterns of pain on workability.^17–19^

Therefore, using the UK Whitehall II Study, we aimed to prospectively examine the determinants of back pain, and consequences of recurrent back pain to exit from paid employment for health and non-health reasons over a prolonged period of the working life span, that is, nearly 30 years. It was possible to include one or more than one health-related or non-health related exits from the workplace for each participant, as participants could also return to work during the follow-up. Socioeconomic factors, obesity and psychosocial working conditions were used as explanatory factors and potential mediators. Their moderating effect was also tested.

## Methods

**Figure 1:**
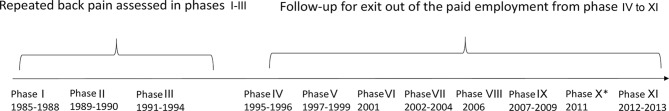
Study design.* Phase X was a pilot among a smaller number of participants testing new measures not used in this study. Further details can be found from the project website (http://www.ucl.ac.uk/whitehallII/data-collection).

Data were from phases I–XI of the British Whitehall II Study, conducted during 1985–2013.^20^ The original source population comprised all civil servants who were working in the London offices of 20 Whitehall departments in 1985–1988, and were aged 35–55 years. The final participants of the study (3413 women and 6895 men) were from clerical and office support grades, middle-ranking executive grades and senior administrative grades. In the current study, phases I–III were the exposure phases while phases IV–XI were used for repeated outcomes (figure 1). Altogether 10 308 employees participated in phase I, of whom 8132 participated in phase II and 8815 in phase III. Of the phase I participants, 1643 were excluded either due to loss to follow-up or non-response to pain questions. Thus, we included participants (n=8665, 69% men, aged 35–55 years at baseline), who had responded at least twice to back pain questions in phases I–III (1985–1994). Participants with back pain at one point in time, and pain at two to three time points were compared with those with no pain. Participants were followed up for their work participation between phases IV and XI (1995–2013).

## Back pain

For the current study, back pain was defined by the response to a question asking participants: ‘Have you had any of the following symptoms in the last fourteen days’. An item in the list of symptoms was ‘backache or pain in the back’, and the response alternatives were ‘yes’ or ‘no’. This variable was similarly measured in phases I–III (1985–1994). The exposure was classified as follows: no pain at any of the phases (reference); a single pain report; one and two or three pain reports (recurrent pain). The variable was also used cumulatively, that is, as a sum of all pain reports (0–3) in phases I–III.

## Outcomes

Data about work participation and transition(s) out of paid employment were self-reported in the follow-up surveys in 1995–2013 (phases IV–XI). The information allowed us to determine transitions out of paid employment for different reasons (unemployed, retired, retired for health reasons, long-term sick, home maker, student and other), and retain employed people as a reference group. Each phase that was preceded by a phase where a participant had been employed was included in the analysis. Thus, we could allow for participants who left employment and subsequently returned to work during the follow-up. At each follow-up phase, participants were classified as ‘employed’, if they were in paid employment either in the civil service or elsewhere. Based on the responses, and following previous procedures,^21^ routes of exit from paid employment were classified into the following categories: ‘health-related exit’ comprising people on long-term sick leave or retired on health grounds; ‘non-health related exit’ comprising all other participants reporting that they were retired; ‘exit due to unemployment’; and ‘other exit’, comprising home makers, students and other reasons for not working. For the civil servants in the Whitehall II Study, the occupational pension age was 60 years for both men and women, and the State Pension age at the time of this study was 65 years for men and 60 years for women. Civil servants also had an option for early retirement or working beyond occupational pension age. All participants were followed-up, including those who changed jobs or who left paid employment.

## Covariates

As work exit is strongly related to age, adjustment for current age was done by fitting a piecewise model (with knots at ages 55 years, 60 years, 65 years) as well as an indicator at age 60 years and 65 years to allow for step changes at occupational pension age, which was 60 years in the civil service, and state pension age, which was 65 years for men and 60 years for women at the time of this study. The models were adjusted for baseline covariates: sex, age and study phase, as well as for self-reported occupational group (low-grade=clerical/support, middle-grade=professionals/ executives, high-grade=administrative personnel), parental education (highest of either parent: low, intermediate, high), body mass index (computed from measured height and weight, in kg/m^2^), high job demands (cut point was the highest quintile of the sum score) and low job control (cut point was the lowest quintile of the sum score). Own education was used in sensitivity analyses (high, intermediate, low). Information about covariates was collected in phase I, except job demands and job control were obtained from phase III.

## Statistical analyses

First, descriptive statistics were computed. Second, we used ordinal logistic regression models to produce cumulative ORs (CORs) for back pain (0, 1, ≥2 reports) by baseline sociodemographic factors. We tested the assumption of COR that the covariate increases the risk similarly for pain reports from 0 to 1 and from 1 to ≥2 and found that it held. Third, our main analysis examined routes of exit from paid employment. Repeated measures logistic regression models were fitted to examine whether the number of back pain reports is a predictor of exit out of paid employment, distinguishing between different routes of exit. Model 1 was adjusted for sex, age and study phase, while model 2 was adjusted for all covariates: sex, age, study phase, parental education, occupational grade, body mass index and psychosocial working conditions.

In an additional analysis, CORs for back pain by baseline sociodemographic factors were calculated by sex, since prevalence of pain and risk of exit differ between women and men. In further analysis, the models were additionally adjusted for participants’ educational attainment to confirm the contribution of socioeconomic position to the associations. Educational attainment was only used in the additional analysis as it was measured only in phase V and had many missing values. We also tested interactions between pain and the possible confounders and mediators (sex, occupational grade, obesity, and working conditions) for the exit route that had the strongest associations with back pain. To confirm whether early pain is merely a predictor of later pain, we performed a sensitivity analysis where we stratified the analyses by pain reports at phase VII. In an additional sensitivity analysis we ran Cox survival models for the first and last exit by the different exit routes. SAS statistical software, V.9.4 was used for all the analyses.

## Results

Of the participants, 961 were still working at the last study phase, while 1012 had two or more exits. During the follow-up there were in total 2330 health-related exits, 474 exits due to unemployment, 1036 exits due to other reasons and 6038 non-health related exits. By the follow-up phases the total numbers of exits (n, non-responders) were: 2683 (48), 1978 (87), 1548 (139), 1260 (15), 1267 (87), 726 (195) and 416 (326) (total exits 9878).

Distributions of baseline (phase I) covariates by number of back pain reports in phases I–III are displayed in table 1. While the population comprised more men than women, recurrent pain was more prevalent among women (table 1). Recurrent pain was also more likely among participants: working at a low occupational grade; reporting low job control; and whose parents had lower educational attainment (all p values<0.05).

**Table 1:** Distribution of baseline (phase I) covariates by back pain in phases I–III (n=8865)

Covariate *(n missing)*	Number of phases with reported back pain*		
	0	1	≥2
Sex *(0)*	N (%)		
Male	3604 (61)	1447 (24)	891 (15)
Female	1283 (47)	782 (29)	658 (24)
Age (0), years			
35–39	1381 (59)	585 (25)	366 (16)
40–49	2267 (56)	1035 (26)	743 (18)
≥50	1239 (54)	609 (27)	440 (19)
Occupational grade *(0)*			
High (administrative)	1642 (62)	633 (24)	374 (14)
Middle (professionals/executive)	2404 (57)	1087 (26)	749 (18)
Low (clerical/support)	841 (47)	509 (29)	426 (24)
Parents’ education *(914)*			
High	960 (61)	356 (23)	262 (17)
Intermediate	864 (56)	438 (28)	250 (16)
Low	2560 (55)	1204 (26)	857 (19)
Body mass index *(11)*			
Recommended weight	3068 (58)	1332 (25)	915 (17)
Overweight	1533 (55)	731 (26)	526 (19)
Obese	281 (51)	162 (30)	106 (19)
Job control *(72)*			
High	4015 (57)	1787 (26)	1192 (17)
Low	1402 (53)	696 (26)	536 (20)
Job demands *(44)*			
Low	4084 (57)	1812 (25)	1270 (18)
High	783 (54)	404 (28)	268 (18)

*P values (χ^2^): cross-tabulations between covariates and number of pain reports were all significant (p values<0.05) except borderline for job demands (0.07).

The likelihood of pain (COR of back pain) by covariates is displayed in table 2. Women had a higher likelihood than men to report pain at one or two to three occasions (COR of repeated back pain for women vs men 1.73; 95% CI 1.59 to 1.89). CORs of back pain were also slightly higher for older versus younger participants, and for lower versus highly educated participants. A similar pattern was seen for parental education, with participants whose parents had intermediate or low levels of education being more likely to report pain than participants whose parents were highly educated. There was also a gradient by occupational grade, that is, the likelihood of repeated back pain was higher for middle-grade than high-grade employees, while the cumulative odds were the highest for the lower-grade employees. Furthermore, high job demands were associated with a higher cumulative likelihood of pain. In addition, being overweight or obese slightly increased the COR for back pain.

**Table 2:** Cumulative ORs (CORs, 95% CIs) for back pain by baseline sociodemographic factors, body mass index and psychosocial working conditions

Covariate/factor	Back pain	
	COR*	95% CI
Sex (n=8665)		
Male	1	
Female	1.73	1.59 to 1.89
Age (years, n=8665)		
30–39	1	
40–49	1.13	1.03 to 1.25
≥50	1.18	1.06 to 1.33
Occupational grade (n=8665)		
High (administrative)	1	
Middle (professionals/executive)	1.20	1.09 to 1.33
Low (clerical/support)	1.39	1.22 to 1.59
Parents’ education (n=7751)		
High	1	
Intermediate	1.21	1.06 to 1.39
Low	1.19	1.07 to 1.34
Body mass index (n=8654)		
Recommended weight	1	
Overweight or obese	1.14	1.04 to 1.24
Job control (n=8593)		
High	1	
Low	1.06	0.95 to 1.18
Job demands (n=8621)		
Low	1	
High	1.19	1.07 to 1.33

*Adjusted for sex and age.

In the additional analyses, the CORs were examined separately for women and men (supplementary file, online supplementary table S1). The associations were broadly similar, although the strength of the associations varied. However, no interactions by sex were found.

10.1136/oemed-2018-105202.supp1 Supplementary file 1

Table 3 shows the ORs for the different routes of exit from paid employment by the number of pain reports during phases I–III. Reporting pain only on a single occasion did not increase the likelihood of exit from paid employment for any reason. In contrast, people who reported pain at least twice were more likely to exit employment for any reason during follow-up (sex, age and phase-adjusted OR 1.14; 95% CI 1.05 to 1.25). Full adjustment for all the covariates had a negligible effect on the association.

**Table 3:** ORs (95% confidence intervals) for exit from paid employment due to any cause, non-health-related reasons, health reasons, unemployment and other reasons by the history of back pain

Number of phases with reported back pain	Exit from paid employment			
	Model 1*		Model 2†	
Exit for any cause	OR	95% CI	OR	95% CI
Back pain 1 vs 0 times	1.07	0.99 to 1.15	1.06	0.97 to 1.15
Back pain ≥2 vs 0 times	1.14	1.05 to 1.25	1.14	1.04 to 1.25
Non-health-related exit				
1 vs 0	1.03	0.92 to 1.11	1.01	0.92 to 1.11
≥2 vs 0	1.07	0.96 to 1.20	1.07	0.96 to 1.21
Health-related exit				
1 vs 0	1.20	0.96 to 1.50	1.15	0.89 to 1.49
≥2 vs 0	1.90	1.50 to 2.41	1.51	1.15 to 1.99
Exit for unemployment				
1 vs 0	1.09	0.84 to 1.40	1.12	0.84 to 1.50
≥2 vs 0	1.13	0.84 to 1.51	1.07	0.76 to 1.50
Exit for other reasons				
1 vs 0	1.03	0.82 to 1.29	1.12	0.87 to 1.43
≥2 vs 0	0.99	0.76 to1.29	1.08	0.81 to 1.44

*Model 1 adjusted for sex, age and study phase.

†Model 2 adjusted for sex, age, study phase, occupational grade, parental education, body mass index, job demands and job control.

Recurrent back pain was most strongly associated with exit from employment due to health reasons (sex, age and phase-adjusted OR 1.90; 95% CI 1.50 to 2.41). This association was somewhat attenuated after adjustment for occupational grade, parental education, job demands, job control and body mass index (OR 1.51; 95% CI 1.15 to 1.99). However, back pain did not significantly increase the risk of exit through other routes (non-health related exit, exit due to unemployment or other (exit) reasons) (table 3). Additional adjustment for the participant’s own educational attainment produced practically identical results to those of the main analyses (data not shown).

In further analyses (data not shown), differences in exit due to health reasons by subgroups were statistically non-significant. However, a gradient was observed for occupational grade so that the associations were stronger for those with lower-grade occupations and those who reported low job control. Thus, when back pain was modelled as a continuous variable, both middle-grade employees (OR 1.30; 95% CI 1.11 to 1.53) and low-grade employees (OR 1.28; 95% CI 1.06 to 1.55) had a higher likelihood of exit due to health reasons, than high-grade employees.

Results of the sensitivity analyses are stratified by pain reports in phase VII and shown in supplementary file, online supplementary table S2. Due to selection issues (many participants had already exited due to health reasons before phase VII) and low numbers, the results should be interpreted with caution. The patterns of the associations were, nonetheless, similar to those in the main analyses, supporting the main finding about the contribution of recurrent pain to subsequent exit from paid employment.

Survival analyses confirmed our findings indicating largest HRs for the first and last exits due to health reasons among those with two or more pain reports (supplementary file, online supplementary tables S3 and S4).

**Figure 2:**
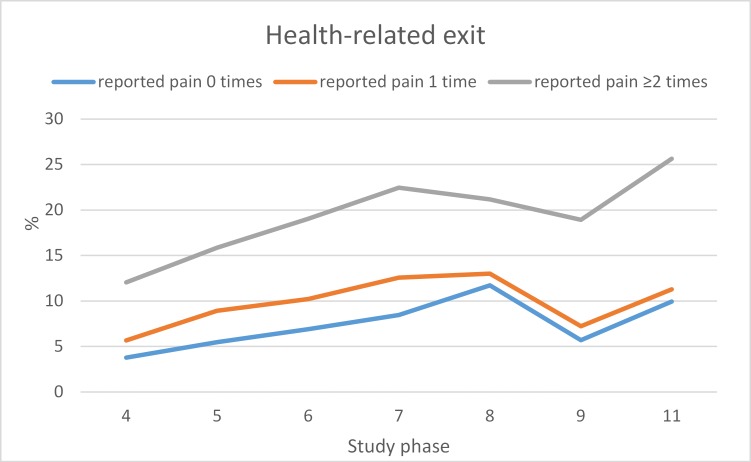
Proportions of participants (%) over the study phases who left paid employment for health reasons, by back pain at baseline.

Recurrent pain had the clearest link to health-related exit. Figure 2 displays the proportion of participants leaving paid employment due to health-related reasons by the number of back pain reports and study phase. The figure shows that over time, the proportion of participants who exited work for health-related reasons did not markedly differ between those reporting back pain only on a single occasion and those who did not report back pain. However, the proportion of participants who left paid employment due to health reasons and who reported pain at least twice, increased at nearly each follow-up phase (from phase IV to phase XII), over a period of nearly a couple of decades.

## Discussion

This study examined recurrent back pain as a predictor of different routes of exit out of paid employment over a follow-up of almost a couple of decades after the pain reports. The main finding was that recurrent back pain was associated with higher odds of exiting from paid work for health-associated reasons. The effects were stronger among employees doing lower-grade and middle-grade, rather than high-grade, work but working conditions did not act as effect modifiers. Pain at a single time point did not increase the likelihood of exit, and recurrent pain did not increase the likelihood of exit for non-health-related reasons.

Other investigators have found an association of chronic pain with health-related exit from the workforce.^2^ However, it is a particular strength of the current study that data about back pain were collected prospectively at three time points and the outcome measure of exit from employment was also collected prospectively with no reliance on recall of previous back pain. In most other studies, pain has been measured at a time point and classified as ‘acute’ (<3 months) or ‘chronic’ (lasting >3 months) based on the response. Thus, the current study, showing that recurrent back pain is a risk factor for exit out of paid employment for health reasons, is novel and extends and complements previous evidence. Our findings may suggest that if pain is measured only once, the effects of low back pain may be attenuated and/or reflect the effects of recurrent pain. A recent study showed that people with chronic pain are, in addition to having a higher risk of health-related exit, more likely to lose their jobs than their healthy counterparts.^22^ However, that study was small-scale and cross-sectional, and causal inferences cannot be drawn. Importantly, there is evidence from a systematic review of intervention studies that individually focused interventions can reduce the risk of health-related exit and job loss, despite having little impact on pain itself.^23^ Together, these findings suggest that recurrent back pain is an important risk factor for future exit out of paid employment justifying the development and use of supportive measures to help people continue working despite pain.

As has been reported elsewhere,^24^ we found that women had a higher prevalence of back pain (a report and recurrent reports) than men but there were no gender differences in the associations with job exit. Moreover, the overall contribution of covariates to the association was relatively minor. For example, we did not observe that obesity or adverse working conditions contributed to the association between pain and the risk of exit out of paid employment. We found that overweight and obesity were risk factors for recurrent low back pain in this study and in another large meta-analysis,^25^ and contributed to the risk of health-related exit due to disability,^26^ but that their effect on the *association* between pain and early exit seemed to be minor. Our group recently explored the interplay of working conditions, ill-health and health-related exit, and found no mitigating effect of working conditions on workforce exit for the chronic diseases studied.^21^ However, the chronic diseases addressed in the other study did not include pain or musculoskeletal disorders

Although with several strengths, this study had some limitations that need to be acknowledged. First, the data were self-reported, both regarding the exposure and outcome, which generally could raise questions about common method bias. However, in our case, it is difficult to see how reporting pain would increase reporting exit out of paid employment particularly given the long duration between data collection for the exposure and outcome. There is no particular reason to assume that people would not correctly report whether they are still in paid employment or have exited, and moreover, why this would differ by pain reports during earlier study phases. Furthermore, as experience of pain is subjective, the presence or absence of pain can only be obtained through self-report^27^ but we cannot rule out that pain could be perceived and/or reported differently by individuals in different occupational groups.^28^ A previous study showed that lower-grade employees had more work disability due to back pain, and that the effects of job control on work disability varied in their magnitude and direction by occupational grade.^29^ These differences in perceived pain and working conditions could explain, at least to some extent, the observed differences in the likelihood of exit between people in different occupational grades in our study.

Second, another caveat is that we could not account for physical workload, which has been shown to have a consistent effect on back pain in both sexes,^28 30^ while psychosocial working conditions may more strongly relate to back pain in men.^28^ Since all our participants were white-collar employees at baseline, physical workload is a relatively unlikely contributor to pain in this cohort. However, it is plausible that civil service workers may differ in some work exposures, for example, posture, ergonomic factors and the amount of sedentary work, and we were unable to allow for this in the current study. The role of psychosocial working conditions in pain has been studied quite extensively, but even though their contribution to back pain and work exit has been supported, their role appears small and the associations are less consistent than for physical working conditions.^23 31 32^ Some prior evidence suggests that differences in psychosocial factors may distinguish between people with chronic pain, and contribute to who can and cannot continue working despite pain.^31^ There are numerous ways of assessing psychosocial working conditions reflected by models such as effort-reward imbalance and demand-control support. Not all factors were measured in the current study but neither high job demands nor low job control modified the association between repeated pain and health-related exit.

Third, healthy worker effect and selection should be considered.^33^ The follow-up covered roughly a couple of decades, and we might have lost participants over time due to health-related attrition. As all the participants were already in mid-life at baseline, and were likely to have a long working history, we cannot rule out that there could already have been health-related selection before the beginning of the study. A non-response and attrition analysis has shown that the mortality hazard is doubled among non-respondents and those who miss some of the follow-up phases.^34^ Nonetheless, the study design and the analyses allowed us to estimate the risk of exit over a time period of 20 years. This means that if health-related exit occurred in an early study phase that was also captured as the participants did not need to have participated at all 11 phases. If health-related selection had an effect, it would more likely have made the results more conservative.

Fourth, some uncertainty remains when using early pain as a predictor of an early exit. As early pain predicts later pain, this suggests that the observed associations could be mediated through continuous pain. If that were the case, early pain would act as a marker of later pain. However, our sensitivity analysis did not support this. In addition, our design allowed us to examine the effects of pain on exit at each time point after baseline, at the end of the follow-up. One could further assume that employees who continue to work despite pain are more resilient, have had work modifications to help them continue working, or perhaps that those with the most severe pain might have exited the earliest. The latter has been suggested before.^35^ If we only included those with continuous pain throughout the follow-up, the population would have become very selective, and the true effects could not be seen. Finally, we acknowledge that some residual, unmeasured confounding likely remained which could account for some of the associations.

To conclude, recurrent back pain was a marker of the risk of exit from paid employment for health reasons. Measuring pain only once may therefore not be sufficient to capture the associations between pain and work exit, and it is likely that earlier studies measuring pain only at a time point have thus underestimated the risk. Further intervention or other studies could examine whether modification of work exposures or other risk factors of pain could reduce the risk of exit due to pain, and help extend work participation. Since we had a male-dominated cohort comprising only white-collar civil servants, further research is needed from the private sector as well as among manual workers, where the prevalence of pain, and subsequent risk of early exit out of paid employment, can be assumed to be even higher. As this is an epidemiological study, direct implications for clinical practice are conjectural, but our findings suggest that it is possible that strategies to identify and prevent recurrent cases of back pain or to make workplace adjustments for those with recurrent back pain could reduce the risk of exit from work and thereby make an impact on the overall burden of musculoskeletal disorders on work disability.
