# Supplementary material for: Recurrent back pain during working life and exit from paid employment: a 28-year follow-up of the Whitehall II Study
Source: Occup Environ Med. 2018 Oct 4;75(11):786–91. doi: 10.1136/oemed-2018-105202 (PMC6227793; doi:10.1136/oemed-2018-105202)
Supplement: Supplementary file 1 [file oemed-2018-105202supp001.docx]

Supplementary file

**Recurrent back pain during working life and exit from paid employment: a 28-year follow-up of the Whitehall II study**

**Running head:** Recurrent pain and exit from paid employment

Tea Lallukka,^1,2^ Minna Mänty,^2,3^ Cyrus Cooper,^4,5^ Maria Fleischmann,^6^ Anne Kouvonen,^7-9^ Karen E Walker-Bone, ^10^ Jenny Head,^6^ Jaana I Halonen^1^

^1^ Finnish Institute of Occupational Health, Helsinki & Kuopio, Finland

^2^ Department of Public Health, University of Helsinki, Helsinki, Finland

^3^ Department of Research, Development and Innovation (RDI), Laurea University of Applied Sciences, Vantaa, Finland

^4^ MRC Lifecourse Epidemiology Unit, University of Southampton, UK

^5^ NIHR Musculoskeletal Biomedical Research Unit, University of Oxford, Oxford, UK.

^6^ Department of Epidemiology and Public Health, University College London, London, UK

^7^ Faculty of Social Sciences, University of Helsinki, Helsinki, Finland

^8^ SWPS University of Social Sciences and Humanities in Wroclaw, Wroclaw, Poland

^9^ UKCRC Centre of Excellence for Public Health, Queen’s University Belfast, Belfast, UK

^10^ Arthritis Research UK/MRC Centre for Musculoskeletal Health and Work, University of Southampton, Southampton, UK

**Table S1**. Cumulative odds ratios (CORs, 95% confidence intervals) for back pain by baseline covariates.

|  | Women | | | Men | | |
| --- | --- | --- | --- | --- | --- | --- |
|  | COR | 95% CI | | COR | 95% CI | |
| Age (years) |  |  |  |  |  |  |
| 35-39 | 1 |  |  | 1 |  |  |
| 40-49 | 1.22 | 1.02 | 1.46 | 1.10 | 0.97 | 1.24 |
| ≥50 | 1.32 | 1.09 | 1.61 | 1.12 | 0.97 | 1.29 |
| Occupational grade |  |  |  |  |  |  |
| High (Administrative) | 1 |  |  | 1 |  |  |
| Middle (Professionals/ executive) | 1.53 | 1.20 | 1.95 | 1.16 | 1.04 | 1.29 |
| Low (Clerical/ support) | 1.88 | 1.48 | 2.39 | 1.16 | 0.95 | 1.40 |
| Parents’ education |  |  |  |  |  |  |
| High | 1 |  |  | 1 |  |  |
| Intermediate | 1.37 | 1.06 | 1.76 | 1.15 | 0.98 | 1.36 |
| Low | 1.28 | 1.05 | 1.57 | 1.15 | 1.00 | 1.33 |
| Body mass index |  |  |  |  |  |  |
| Recommended weight | 1 |  |  | 1 |  |  |
| Overweight or obese | 1.32 | 1.14 | 1.52 | 1.05 | 0.95 | 1.17 |
| Job control |  |  |  |  |  |  |
| High | 1 |  |  | 1 |  |  |
| Low | 1.21 | 1.01 | 1.44 | 1.10 | 0.98 | 1.22 |
| Job demands |  |  |  |  |  |  |
| Low | 1 |  |  | 1 |  |  |
| High | 1.19 | 1.03 | 1.38 | 1.02 | 0.90 | 1.14 |

**Table S2.** Odds ratios for exit from the paid employment due to any cause, health reasons, non-health related exit and other exit by history of back pain.

| **Number of phases reported back pain** | **Exit from the paid employment** | | | | | |
| --- | --- | --- | --- | --- | --- | --- |
|  | Model 1 | | | Model 2 | | |
| Exit for any cause | OR | 95% CI | | OR | 95% CI | |
| **Sensitivity analysis including those *with back pain* at phase 7*** |  |  |  |  |  |  |
| Exit for health reasons |  |  |  |  |  |  |
| 1 vs. 0 | 1.14 | 0.78 | 1.67 | 1.09 | 0.71 | 1.66 |
| ≥2 vs. 0 | 1.45 | 1.00 | 2.10 | 1.17 | 0.78 | 1.78 |
| without education |  |  |  |  |  |  |
|  |  |  |  |  |  |  |
| **Sensitivity analysis including those *without back pain* at phase 7*** |  |  |  |  |  |  |
| 1 vs. 0 | 1.02 | 0.71 | 1.48 | 1.00 | 0.65 | 1.54 |
| ≥2 vs. 0 | 1.66 | 0.97 | 2.86 | 1.36 | 0.77 | 2.41 |

Model 1 adjusted for sex, age, and study phase

Model 2 adjusted for sex, age, study phase, occupational status, parental education, body mass index, job demands, and job control

**Table S3.** Hazard ratios (95% CI) for the first exit from paid employment in relation to back pain.

| **Number of phases reported back pain** | **Exit from paid employment** | | | | | |
| --- | --- | --- | --- | --- | --- | --- |
|  | Model 1^a^ | | | Model 2^b^ | | |
| Exit for any cause | HR | 95% CI | | HR | 95% CI | |
| Back pain 1 vs. 0 times | 1.07 | 1.01 | 1.14 | 1.07 | 1.00 | 1.14 |
| Back pain ≥2 vs. 0 times | 1.21 | 1.13 | 1.30 | 1.20 | 1.11 | 1.30 |
|  |  |  |  |  |  |  |
| Non-health related exit |  |  |  |  |  |  |
| 1 vs. 0 | 1.04 | 0.97 | 1.11 | 1.03 | 0.96 | 1.11 |
| ≥2 vs. 0 | 1.13 | 1.05 | 1.23 | 1.15 | 1.05 | 1.25 |
|  |  |  |  |  |  |  |
| Health-related exit |  |  |  |  |  |  |
| 1 vs. 0 | 1.39 | 1.12 | 1.72 | 1.31 | 1.03 | 1.67 |
| ≥2 vs. 0 | 2.08 | 1.68 | 2.57 | 1.85 | 1.45 | 2.35 |
|  |  |  |  |  |  |  |
| Exit for unemployment |  |  |  |  |  |  |
| 1 vs. 0 | 1.11 | 0.86 | 1.43 | 1.16 | 0.87 | 1.54 |
| ≥2 vs. 0 | 1.21 | 0.91 | 1.60 | 1.19 | 0.86 | 1.65 |
|  |  |  |  |  |  |  |
| Exit for other reasons |  |  |  |  |  |  |
| 1 vs. 0 | 1.04 | 0.84 | 1.30 | 1.13 | 0.89 | 1.43 |
| ≥2 vs. 0 | 0.98 | 0.76 | 1.27 | 1.01 | 0.76 | 1.35 |

**Table S4.** Hazard ratios (95% CI) for the last exit from paid employment in relation to back pain.

| **Number of phases reported back pain** | **Exit from paid employment** | | | | | |
| --- | --- | --- | --- | --- | --- | --- |
|  | Model 1^a^ | | | Model 2^b^ | | |
| Exit for any cause | HR | 95% CI | | HR | 95% CI | |
| Back pain 1 vs. 0 times | 1.38 | 1.11 | 1.71 | 1.32 | 1.03 | 1.67 |
| Back pain ≥2 vs. 0 times | 1.98 | 1.59 | 2.46 | 1.71 | 1.33 | 2.19 |
|  |  |  |  |  |  |  |
| Non-health related exit |  |  |  |  |  |  |
| 1 vs. 0 | 1.05 | 0.97 | 1.13 | 1.05 | 0.97 | 1.14 |
| ≥2 vs. 0 | 1.03 | 0.94 | 1.12 | 1.04 | 0.95 | 1.14 |
|  |  |  |  |  |  |  |
| Health-related exit |  |  |  |  |  |  |
| 1 vs. 0 | 1.23 | 0.96 | 1.57 | 1.22 | 0.92 | 1.60 |
| ≥2 vs. 0 | 1.92 | 1.52 | 2.44 | 1.80 | 1.38 | 2.34 |
|  |  |  |  |  |  |  |
| Exit for unemployment |  |  |  |  |  |  |
| 1 vs. 0 | 0.94 | 0.65 | 1.37 | 0.98 | 0.64 | 1.49 |
| ≥2 vs. 0 | 1.25 | 0.85 | 1.83 | 1.24 | 0.81 | 1.90 |
|  |  |  |  |  |  |  |
| Exit for other reasons |  |  |  |  |  |  |
| 1 vs. 0 | 1.05 | 0.79 | 1.39 | 1.18 | 0.87 | 1.60 |
| ≥2 vs. 0 | 0.97 | 0.70 | 1.35 | 0.99 | 0.69 | 1.42 |
